# Immune Profiles Elicited by Different HBV Vaccine Modalities and Their Associations with Serological and Intrahepatic Viral Outcomes

**DOI:** 10.3390/ijms27198499

**Published:** 2026-09-23

**Authors:** Huiying Xiao, Jinhang He, Lili Zhu, Songmei Wang, Chao Qiu, Xuanyi Wang

**Affiliations:** 1 Institutes of Biomedical Sciences, Fudan University, Shanghai 200032, China; 2 Key Laboratory of Medical Molecular Virology (MOE/NHC/CAMS), Shanghai Medical College, Fudan University, Shanghai 200032, China; 3 Shanghai Institute of Infectious Disease and Biosecurity, Fudan University, Shanghai 200032, China; 4 Department of Infectious Diseases, Huashan Hospital, Fudan University, Shanghai 200040, China; 5 Laboratory of Medical Molecular Biology, Experimental Teaching Center, School of Basic Medical Sciences, Fudan University, Shanghai 200032, China; 6 Minhang Hospital, Fudan University, Shanghai 201199, China

**Keywords:** hepatitis B virus, antigen–antibody immune complex, DNA vaccines, CD8^+^ T-cell responses

## Abstract

Therapeutic vaccination for chronic hepatitis B aims to restore HBV-specific T-cell immunity and contribute to a functional cure. We systematically compared the immune profiles and antiviral efficacy of four vaccine modalities: recombinant HBsAg (rHBsAg), antigen–antibody immune complex (IC), a DNA vaccine encoding HBsAg (DNA-HBsAg), and an HBsAg-Fc fusion DNA vaccine (DNA-HBsAg-Fc). In naïve mice, the rHBsAg vaccine predominantly induced anti-HBs IgG responses, whereas the IC vaccine elicited strong anti-HBs IgG and detectable CD4^+^ T-cell responses. In contrast, DNA vaccines preferentially induced HBsAg-specific CD8^+^ T-cell immunity, which was further enhanced by fusion to the Fc fragment of human IgG1. In the pre-immunization/AAV-HBV model, high anti-HBs titers in rHBsAg and IC groups were associated with rapid clearance of circulating HBsAg but not with comparable reductions in HBcAg^+^ hepatocytes, whereas stronger HBsAg-specific CD8^+^ T-cell responses in the DNA vaccine groups were associated with greater reductions in HBcAg^+^ hepatocytes. However, in the therapeutic AAV-HBV model, all vaccine monotherapies showed limited and non-sustained antiviral efficacy, with only transient reductions in serum HBsAg and limited reductions in HBcAg^+^ hepatocytes. These findings highlight the distinct immune profiles of different vaccine platforms and an association between stronger HBsAg-specific CD8^+^ T-cell responses and reduced intrahepatic HBcAg expression, while underscoring the limited efficacy of vaccine monotherapy in the AAV-HBV model.

## 1. Introduction

Chronic hepatitis B (CHB), caused by persistent hepatitis B virus (HBV) infection, remains a major public health burden in China [1]. HBV-specific CD8^+^ T cells play a critical role in the control and clearance of HBV infection. In a chimpanzee model of acute HBV infection, liver-infiltrating CD8^+^ T cells were shown to secrete antiviral cytokines, including IFN-γ, thereby promoting the clearance of HBV DNA and RNA from infected hepatocytes primarily through noncytolytic mechanisms, with limited destruction of infected cells [2,3]. In acute self-limiting HBV infection, robust HBV-specific CD8^+^ T-cell responses contribute to spontaneous viral clearance and the establishment of durable immunological memory [4,5]. Conversely, in patients with CHB, these responses are profoundly impaired and are scarcely detectable even after 10–14 days of in vitro peptide expansion [6,7]. Although standard-of-care medicines, including type I interferons and nucleos(t)ide analogues (NAs), effectively suppress viral replication, alleviate liver injury, and delay disease progression, they could only achieve a functional cure in a small proportion of patients and generally fail to restore HBV-specific T-cell immunity [8,9,10]. Therefore, restoring vigorous and functional HBV-specific CD8^+^ T-cell immunity represents a key immunological rationale for the development of novel curative immunotherapies for CHB.

Therapeutic vaccines represent a promising strategy for achieving a functional cure. To this end, Wen et al. developed an innovative antigen–antibody immunogenic complex (IC) vaccine comprising yeast-derived HBsAg and hepatitis B immune globulin (HBIG) at an optimal ratio, together with additional equivalent amount of free HBsAg to improve solubility and immunogenicity, and formulated with an aluminum-based adjuvant [11,12]. By engaging Fcγ receptors (FcγRs) on antigen-presenting cells (APCs) via the antibody Fc domain, the IC facilitates antigen uptake and cross-presentation, thereby promoting both humoral and cellular immune responses [13]. Importantly, the IC vaccine has been reported to skew the cellular immunity toward a Th1 phenotype, characterized by IFN-γ and IL-2 production, which is closely associated with antiviral immune responses [14]. However, the further clinical development and application of this protein-based IC vaccine have been limited by challenges related to manufacturing, batch variation in raw-materials.

DNA vaccines drive endogenous antigen expression in APCs, priming antigen-specific CD8^+^ T cells via the MHC class I pathway. Simultaneously, secreted antigens can be taken up by APCs and presented to CD4^+^ T cells via MHC class II molecules. These complementary antigen-presentation pathways enable DNA vaccines to induce both cellular and antibody responses, supporting their potential as therapeutic vaccine platforms [15]. However, the immune profiles and antiviral efficacy across these distinct vaccine modalities—recombinant protein, IC, and DNA vaccines—have not been systematically compared.

To circumvent the limitations of the protein-based IC vaccine and explore whether Fc fusion could enhance antigen presentation and cellular immunogenicity in a DNA vaccine platform, we constructed two DNA vaccines encoding either HBsAg alone (DNA-HBsAg) or an HBsAg-human IgG1 Fc fusion protein (DNA-HBsAg-Fc). Their immunogenicity and immune profiles were compared with those of the recombinant HBsAg (rHBsAg) and IC vaccines in naïve mice. We further evaluated their antiviral efficacy in both a pre-immunization/AAV-HBV transduction model and a therapeutic AAV-HBV model. In this study, we sought to establish the key immunological clues of intrahepatic HBV eradication and to define the limitations of vaccine monotherapy in the context of chronic immune tolerance, thereby providing a rationale for the development of next-generation therapeutic vaccines against CHB.

## 2. Results

### 2.1. Construction and In Vitro Characterization of DNA Vaccines

We constructed two DNA vaccines by inserting sequences encoding either HBsAg (DNA-HBsAg) or HBsAg fused to human IgG1 Fc through a (G4S)_2_ linker (DNA-HBsAg-Fc) into the pVAX1 plasmid (Figure 1A,B). To characterize their expression in vitro, HEK293T cells were transfected with the respective plasmids, and both cell lysates and culture supernatants were analyzed for the expression of designed proteins. Western blot analysis of the cell lysates confirmed the expression of HBsAg (~25 kDa) and the HBsAg-Fc fusion protein (~52 kDa) (Figure 1C). The HBsAg-Fc fusion protein was detected by both anti-HBsAg and anti-human IgG1 Fc antibodies, supporting the presence of both HBsAg and Fc components in the expected ~52 kDa fusion protein. Quantitative HBsAg measurement showed that the intracellular HBsAg level detected in DNA-HBsAg-Fc-transfected cells was significantly lower than that in DNA-HBsAg-transfected cells (Figure 1D). In culture supernatants, HBsAg was readily detectable in DNA-HBsAg-transfected cells, whereas HBsAg-Fc remained below the lower limit of detection (LLoD) of the assay (Figure 1E).

### 2.2. Different Vaccine Modalities Induce Distinct Immune Profiles

To systematically compare the immune profiles elicited by different vaccine modalities, C57BL/6J mice were intramuscularly immunized with the rHBsAg vaccine, the IC vaccine, and the DNA vaccines (DNA-HBsAg-Fc and DNA-HBsAg) at weeks 0, 2, 4, and 6, while the control group received empty pVAX1 (Figure 2A). Both the rHBsAg and IC vaccines induced high levels of anti-HBs IgG responses, significantly surpassing those elicited by the DNA vaccines. Specifically, the anti-HBs geometric mean titers (GMTs) for the rHBsAg and IC vaccines reached 270,235 (95% CI: 56,590–1,290,457) and 188,202 (95% CI: 73,116–4,831,471), respectively, whereas DNA-HBsAg and DNA-HBsAg-Fc yielded GMTs of only 3676 (95% CI: 568.9–23,751) and 75.8 (95% CI: 5.9–973.6), respectively (Figure 2B). These results indicate that, under the immunization conditions used in this study, the rHBsAg and IC formulations elicited substantially higher HBsAg-specific IgG titers than the two DNA vaccines.

In contrast, DNA vaccines preferentially induced cellular immune responses. IFN-γ ELISpot assays of splenocytes stimulated with the HBsAg peptide pool revealed that DNA-HBsAg-Fc elicited significantly higher HBsAg-specific T-cell responses than DNA-HBsAg (307.8 ± 59.8 versus 86.1 ± 25.7 spot-forming cells (SFCs)/10^6^ cells; *p* = 0.0003). The IC vaccine induced a response in comparable magnitude to that of DNA-HBsAg (72.8 ± 10.3 SFCs/10^6^ cells), whereas the rHBsAg vaccine induced negligible T-cell responses (Figure 2C,D). DNA-HBsAg-Fc elicited the strongest overall T-cell response among the vaccine groups, indicating that Fc fusion markedly enhanced the cellular immunogenicity of the DNA vaccine. The stronger response elicited by the IC vaccine relative to rHBsAg further suggested that Fc-mediated antigen targeting may enhance cellular immune responses in protein-based vaccines.

To further characterize the T-cell subsets activated by different vaccine modalities, we analyzed splenocytes stimulated with the HBsAg peptide pool by flow cytometry. The results revealed that the rHBsAg vaccine did not induce appreciable IFN-γ^+^ CD8^+^ or CD4^+^ T-cell responses. In contrast, DNA vaccines primarily induced CD8^+^ T-cell immunity: DNA-HBsAg-Fc induced a significantly higher frequency of IFN-γ^+^ CD8^+^ T cells than DNA-HBsAg (0.30% ± 0.05% versus 0.17% ± 0.04%; *p* = 0.0003), a pattern consistent with the ELISpot results, supporting the ability of IgG1 Fc fusion to enhance HBsAg-specific CD8^+^ T-cell responses. The IC vaccine elicited minimal IFN-γ^+^ CD8^+^ T cells (0.02% ± 0.02%) (Figure 2E and Figure S2). In contrast, within the CD4^+^ compartment, only the IC vaccine induced robust activation, with a significantly higher frequency of IFN-γ^+^ CD4^+^ T cells (0.30% ± 0.06%) than those observed in the DNA-HBsAg-Fc (0.06% ± 0.02%, *p* = 0.0002) and DNA-HBsAg (0.04% ± 0.02%, *p* < 0.0001) groups (Figure 2F and Figure S2). Although IL-2 was included in the intracellular cytokine staining panel, stimulation with the HBsAg peptide pool induced only minimal IL-2 responses in both CD4^+^ and CD8^+^ T cells across all vaccine groups, with frequencies remaining close to background levels. Therefore, IL-2 responses were not subjected to further quantitative comparison.

Furthermore, profiling of memory/effector differentiation based on CD44 and CD62L expression revealed that the activated IFN-γ^+^ CD8^+^ T cells induced by DNA vaccines and the IFN-γ^+^ CD4^+^ T cells induced by the IC vaccine both predominantly exhibited a CD44^+^ CD62L^−^ phenotype. This phenotype is consistent with an effector/effector-memory (Teff/Tem) profile, indicating that the responding T-cell populations induced by these vaccine platforms were predominantly enriched for a Teff/Tem phenotype (Figure 2G,H and Figure S3).

Overall, the rHBsAg vaccine predominantly induced anti-HBs IgG, whereas the IC vaccine elicited both potent anti-HBs IgG and CD4^+^ T-cell responses, with only minimal CD8^+^ T-cell activation. Conversely, DNA vaccines primarily induced robust HBsAg-specific CD8^+^ T-cell immunity, which was significantly enhanced by fusing HBsAg with the IgG1 Fc fragment.

### 2.3. HBsAg-Specific CD8^+^ T-Cell Responses Are Associated with Reduced Intrahepatic HBcAg Expression

The AAV-HBV model utilizes the highly hepatotropic AAV8 serotype to achieve liver-specific transduction, delivering the HBV genome directly into hepatocyte nuclei and thereby bypassing the receptor-mediated entry step of natural HBV infection [16]. Since AAV entry is mediated by the AAV8 capsid rather than HBV envelope proteins, vaccine-induced anti-HBs antibodies cannot prevent AAV transduction in vivo. Leveraging this feature, we established a pre-immunization/AAV-HBV transduction model by immunizing naïve mice before the delivery of AAV-HBV into the hepatocytes (Figure 3A). This model is not designed to assess classical antibody-mediated protection against viral entry; instead, it enables evaluation of whether pre-existing vaccine-induced HBV-specific effector T cells and circulating antibodies can clear produced viral antigens and eliminate infected hepatocytes.

Serum HBV DNA and HBsAg levels at 1-week post-transduction confirmed successful AAV-HBV transduction across all groups (Figure 3B,D). Following AAV transduction, serum HBsAg remained below the LLoD throughout the observation period in the IC and rHBsAg vaccine groups. In contrast, the DNA-HBsAg and DNA-HBsAg-Fc groups showed detectable serum HBsAg at week 1 (8.4 IU/mL and 57.1 IU/mL, respectively), but declined below the LLoD by weeks 5 and 10, respectively (Figure 3B and Figure S4). Before AAV transduction, anti-HBs GMTs were 941,014 (95% CI: 458,006–1,933,397) in the IC group and 235,253 (95% CI: 76,593–722,575) in the rHBsAg vaccine group, approximately 194-fold and 49-fold higher than those in the DNA-HBsAg group (4850; 95% CI: 514.1–45,757), and >9000-fold and >2300-fold higher than those in the DNA-HBsAg-Fc group (100; 95% CI: 6.6–1521) (Figure 3C and Figure S4). Longitudinal analysis using two-way repeated-measures ANOVA indicated that anti-HBs trajectories differed among the vaccine groups over time. The inverse relationship between pre-existing anti-HBs titers and initial serum HBsAg levels is consistent with a role for circulating antibodies in facilitating the rapid clearance of newly produced HBsAg. Notably, as serum HBsAg declined, anti-HBs titers in the DNA vaccine groups showed an upward trend relative to their pre-transduction levels, raising the possibility that sustained antigen expression delivered by AAV-HBV may have boosted pre-existing anti-HBs memory responses. The empty pVAX1 control group maintained stable serum HBsAg without detectable anti-HBs. By week 15, serum HBV DNA declined below the LLoD in all vaccine groups, indicating effective clearance of circulating HBV DNA, whereas levels in the control group remained at 10^4^–10^5^ IU/mL. Thus, in this pre-immunization/AAV-HBV transduction model, all vaccine modalities ultimately achieved effective clearance of circulating HBV DNA and HBsAg despite their markedly different humoral and cellular immune profiles.

Intrahepatic HBcAg expression was assessed by HBcAg immunohistochemistry (IHC) staining of liver sections (Figure 3E,F). Despite serum HBsAg levels remaining below the LLoD throughout the observation period in both the IC and rHBsAg vaccine groups, HBcAg^+^ hepatocytes persisted in the liver to varying degrees. The rHBsAg vaccine group showed only little reduction in the proportion of HBcAg^+^ hepatocytes, with a mean percentage of HBcAg^+^ hepatocytes of 31.7% ± 2.7%, comparable to that of the empty pVAX1 control group (34.7% ± 1.9%). In the IC group, although 2 of 5 mice showed lower proportions of HBcAg^+^ hepatocytes, all animals retained detectable intrahepatic HBcAg expression (mean: 21.6% ± 5.2%). In contrast, HBcAg^+^ hepatocytes were not observed in the examined liver sections of 3 of 5 mice (60%) in the DNA-HBsAg-Fc group, with a mean HBcAg^+^ hepatocyte percentage of 3.9% ± 2.7%. Similarly, no HBcAg^+^ hepatocytes were observed in the examined liver sections of 2 of 5 mice (40%) in the DNA-HBsAg group (mean: 8.5% ± 3.8%). These results indicate an association between vaccine-induced HBsAg-specific CD8^+^ T-cell responses and reduced intrahepatic HBcAg expression, whereas high-titer antibody responses or predominantly CD4^+^ T-cell responses were not associated with comparable reductions in HBcAg^+^ hepatocytes.

### 2.4. Protein and DNA Vaccine Monotherapies Exhibit Limited Therapeutic Efficacy in AAV-HBV Mice

Next, we evaluated the therapeutic efficacy of these vaccines in AAV-HBV mice (Figure 4A). Despite the robust immunogenicity of DNA vaccines in naïve mice and their antiviral efficacy in the pre-immunization/AAV-HBV transduction model, serum HBsAg reductions from baseline in the DNA vaccine groups did not exceed 0.5 log_10_ IU/mL in AAV-HBV mice throughout the observation period and were comparable to those observed in the empty pVAX1 control group, indicating that DNA vaccine monotherapy was insufficient to achieve substantial and sustained HBsAg reduction under immune-tolerant conditions (Figure 4B and Figure S5). Longitudinal analysis using two-way repeated-measures ANOVA indicated that the temporal patterns of serum HBsAg changes differed across the experimental groups. The rHBsAg and IC vaccine groups showed initial declines in serum HBsAg, with maximal reductions of approximately 1.0 and 1.5 log_10_ IU/mL, respectively, followed by rebound during extended observation (Figure 4B and Figure S5). Post hoc comparisons identified significant differences between the IC and pVAX1 groups at weeks 4 and 6, but these differences were not sustained at later time points (adjusted *p* = 0.0099 and *p* = 0.0079, respectively; Table S2). Collectively, these findings indicate that neither protein-based nor DNA vaccines, administered as monotherapies, achieved effective and sustained HBsAg reduction in the AAV-HBV model.

In AAV-HBV mice, vaccine-induced HBsAg-specific T-cell responses were markedly attenuated compared with those observed in naïve mice. Although DNA-HBsAg-Fc and IC elicited detectable T-cell responses than DNA-HBsAg and the rHBsAg vaccine, respectively, none of the treatment groups differed significantly from the empty pVAX1 control group (Figure 4C). To further investigate changes in the liver, we performed HBcAg IHC staining on liver sections. The percentages of HBcAg^+^ hepatocytes in the DNA-HBsAg-Fc (30.4% ± 1.7%; *p* = 0.0044), DNA-HBsAg (32.6% ± 2.1%; *p* = 0.0377), and IC (31.3% ± 1.5%; *p* = 0.0114) groups were significantly lower than that in the empty pVAX1 control group (39.4% ± 1.4%), indicating a partial reduction in the proportion of HBcAg^+^ hepatocytes. However, the magnitude of these reductions was modest, and the proportions of HBcAg^+^ hepatocytes remained above 30% in all three treatment groups. Notably, the proportion in the rHBsAg vaccine group (35.7% ± 0.9%; *p* = ns) was comparable to that of the control group (Figure 4D). Collectively, these findings demonstrate that although the Fc-fusion DNA vaccine and the antigen–antibody complex IC were associated with partial reductions in the proportion of HBcAg^+^ hepatocytes, none of the vaccine monotherapies achieved substantial and sustained antiviral effects within the observation period.

## 3. Discussion

Achieving a functional cure for CHB hinges on breaking immune tolerance and restoring HBV-specific T-cell immunity. In this study, we systematically compared the immune polarization and in vivo antiviral efficacy of four HBV vaccines—rHBsAg, IC, DNA-HBsAg, and DNA-HBsAg-Fc. Both the rHBsAg and IC vaccines induced high-titer anti-HBs antibodies in naïve mice. However, the rHBsAg vaccine elicited only minimal specific T-cell responses, whereas the IC vaccine significantly activated CD4^+^ T cells but induced minimal CD8^+^ T-cell responses. In contrast, DNA vaccines robustly elicited CD8^+^ T-cell responses. These disparate immune profiles likely reflect differences in antigen processing and presentation pathways. By driving endogenous protein expression, DNA vaccines facilitate antigen presentation via the MHC class I pathway, efficiently priming CD8^+^ T cells. Conversely, as an exogenous antigen, the rHBsAg protein is primarily processed and presented through the MHC class II pathway, favoring CD4^+^ T-cell help and humoral immunity. Notably, although both DNA-HBsAg-Fc and the IC vaccine incorporate an Fc component, they induced highly divergent T-cell profiles. Previous studies have shown that IC immunization stimulates IFN-γ, but not IL-4, production in murine splenocytes [13]. Our flow cytometry analysis extended these findings by revealing that the IFN-γ^+^ T cells elicited by IC were predominantly CD4^+^ T cells rather than CD8^+^ T cells. This difference may partly reflect the distinct innate immune and adjuvant environments provided by the two vaccine platforms. Unmethylated CpG motifs inherent in the DNA plasmid backbone act as TLR9 agonists, promoting Th1-biased innate immune signaling and thereby supporting cellular immune responses. In contrast, the aluminum-based adjuvant used in the IC formulation can enhance antigen uptake and presentation but generally favors antibody and CD4^+^ T-cell responses and is relatively inefficient at eliciting strong cross-primed CD8^+^ T-cell responses [17]. Consequently, DNA vaccines, combining endogenous antigen expression with intrinsic immunostimulatory properties, may have a distinct advantage in promoting robust CD8^+^ T-cell immunity.

An additional feature of DNA-HBsAg-Fc was the low extracellular HBsAg signal observed in vitro despite its enhanced HBsAg-specific CD8^+^ T-cell response in vivo. Given the membrane-associated and particle-forming properties of HBsAg, fusion of a relatively large Fc domain may alter antigen folding, intracellular trafficking, particle assembly, or secretion, thereby changing the amount and form of antigen available for immune recognition. Reduced extracellular antigen availability may partly contribute to the relatively weak anti-HBs response elicited by DNA-HBsAg-Fc, whereas altered intracellular retention or trafficking may favor endogenous antigen processing through the MHC class I pathway and contribute to enhanced CD8^+^ T-cell priming. FcγR-mediated antigen uptake and cross-presentation are also plausible mechanisms supported by previous studies [13], but the enhanced cellular immunogenicity of DNA-HBsAg-Fc may not be attributed specifically to an FcγR-dependent mechanism. Further studies using FcγR blockade or Fc variants with impaired FcγR binding, together with direct analysis of antigen localization and processing, will be required to distinguish these possibilities. Taken together, these findings suggest that DNA-HBsAg-Fc was associated with an immune profile characterized by stronger cellular but weaker humoral responses relative to DNA-HBsAg. For a therapeutic HBV vaccine, the enhanced cellular response observed with DNA-HBsAg-Fc may be relevant, as restoration of HBV-specific T-cell immunity is a major immunological objective. Nevertheless, the reduced anti-HBs response represents an important trade-off that should be considered in future optimization of this vaccine design. It should also be noted that IFN-γ responses demonstrate HBsAg-specific T-cell responsiveness but do not fully establish cytotoxic capacity, polyfunctionality, or direct antiviral effector function. Additional analyses of cytotoxic mediators, degranulation, target-cell killing, and intrahepatic T-cell function will be required to more fully define the antiviral functionality of these responses.

In the pre-immunization/AAV-HBV transduction model, although mice pre-immunized with the IC or rHBsAg vaccines maintained serum HBsAg negativity, abundant HBcAg^+^ hepatocytes persisted in the liver. These findings indicate that serological HBsAg clearance does not necessarily reflect the elimination of infected hepatocytes, highlighting that peripheral serological markers alone may incompletely reflect intrahepatic viral activity and residual antigen burden [18,19]. Notably, the empty pVAX1 control mice maintained persistent circulating HBsAg without developing detectable anti-HBs. This finding is consistent with the immunotolerant nature of the AAV-HBV model, in which sustained hepatocyte-derived antigen expression occurs in the absence of the inflammatory and costimulatory signals associated with acute infection [20]. Persistent antigen exposure in the tolerogenic hepatic environment may therefore favor immune tolerance or ineffective humoral priming rather than spontaneous anti-HBs seroconversion. Although high-titer antibodies induced by the IC and rHBsAg vaccines effectively facilitated the clearance of circulating HBsAg, neither humoral immunity nor the CD4^+^ T-cell response elicited by IC was sufficient to achieve substantial clearance of infected hepatocytes. In contrast, the DNA-HBsAg-Fc group, which elicited stronger HBsAg-specific CD8^+^ T-cell responses, showed markedly lower proportions of HBcAg^+^ hepatocytes, demonstrating an association between vaccine-induced HBsAg-specific CD8^+^ T-cell responses and reduced intrahepatic HBcAg expression. However, because liver-infiltrating CD8^+^ T cells were not directly quantified, the present data do not establish a direct causal relationship between CD8^+^ T-cell responses and the reduction in HBcAg^+^ hepatocytes. These results further suggest that humoral or predominantly CD4^+^ T-cell responses alone were not associated with comparable reductions in HBcAg^+^ hepatocytes, whereas stronger HBsAg-specific CD8^+^ T-cell responses were associated with greater reductions in intrahepatic HBcAg expression. Therefore, the induction of robust HBV-specific CD8^+^ T-cell responses remains an important objective for future therapeutic HBV vaccine development.

It should be emphasized that the rAAV8-HBV1.3 system used in this study is a viral vector-mediated HBV transduction model rather than a conventional HBV infection model. Although this model can sustain hepatic HBV antigen expression and recapitulate aspects of persistent antigen exposure and immune tolerance, it does not fully reproduce several key features of natural HBV infection [21]. In particular, cccDNA is not robustly established through the canonical rcDNA repair pathway in AAV-HBV models, and detectable cccDNA may arise through alternative mechanisms, including recombination of AAV-HBV episomes [16]. Moreover, intrahepatic HBV DNA, HBV RNA, and cccDNA were not quantified in the present study. Therefore, HBcAg IHC should be interpreted as a measure of intrahepatic HBV antigen expression rather than as evidence of complete virological eradication. Further validation in complementary animal models that more closely recapitulate natural HBV infection and cccDNA biology will be important for evaluating the translational relevance of these findings.

In the AAV-HBV model, these vaccines elicited partial or transient antiviral effects, but none achieved sustained serum HBsAg clearance, restoration of HBV-specific T-cell responses, or complete loss of detectable intrahepatic HBcAg expression. This limited efficacy may reflect immunological challenges relevant to CHB, particularly persistent antigen exposure and the tolerogenic hepatic microenvironment. HBV infection is generally associated with weak or delayed innate inflammatory responses, while hepatocytes, although capable of presenting antigen through MHC class I molecules, lack the classical costimulatory capacity required for efficient priming of functional CD8^+^ T-cell responses. Consequently, persistent recognition of hepatocyte-expressed antigens by vaccine-primed CD8^+^ T cells can impair their differentiation into effector and memory populations and instead promote dysfunction, apoptosis, clonal deletion, or anergy. The loss or dysfunction of hepatocyte-interacting CD8^+^ T cells may be further reinforced by multiple hepatic tolerance mechanisms, including Kupffer cell-mediated phagocytosis, natural killer cell-mediated killing, and intracellular lysosomal degradation following T-cell invasion into hepatocytes [22,23,24,25,26,27]. Together, these mechanisms create a substantial barrier to the restoration of functional HBV-specific CD8^+^ T-cell immunity during chronic antigen exposure.

Building upon this tolerogenic background, we postulate that the striking discrepancy in efficacy between the AAV-HBV model and the pre-immunization/AAV-HBV transduction model may be explained by two key biological differences. First, the timing of antigen exposure likely dictates the baseline quality of the T-cell pool. In the pre-immunization/AAV-HBV transduction model, HBV-specific T cells are primed in an immunologically intact environment before antigen exposure, allowing the establishment of a vaccine-primed antigen-specific T-cell pool before hepatic antigen expression. Conversely, in the AAV-HBV model, high levels of viral antigens are already present prior to vaccination. We speculate that vaccine-primed CD8^+^ T cells encounter a sustained high antigen burden upon reaching the liver, favoring functional exhaustion or clonal deletion rather than efficient expansion and effector differentiation. Second, the kinetic balance between immune attack and antigen burden is reversed. Pre-existing vaccine-primed T cells can respond immediately as hepatic antigen expression emerges following AAV-HBV transduction in the pre-immunization/AAV-HBV transduction model, whereas T cells generated by therapeutic vaccination must confront an already established and sustained antigen burden in the AAV-HBV model. Under these conditions, the magnitude and quality of the immune response induced by vaccine monotherapy may be insufficient to overcome pre-existing T-cell dysfunction and persistent antigenic stimulation. This interpretation is consistent with clinical observations: the therapeutic DNA vaccine JNJ-6430535 induced HBV-specific T-cell responses in 92% of healthy volunteers but in only 50% of patients with CHB [28].

Of note, the DNA and protein-based vaccines in this study were administered using platform-specific dosing regimens rather than dose-matched regimens. Therefore, the differences observed among these vaccine modalities should be interpreted as comparisons of the immune profiles elicited by the respective platforms under the tested conditions, rather than as direct dose-equivalent comparisons of vaccine potency. In addition, the DNA vaccines were administered intramuscularly as naked plasmid DNA at a high dose (100 μg per dose). Because the present study was designed primarily to compare the immunological profiles of the vaccine constructs rather than to optimize DNA delivery, electroporation or other delivery-enhancing technologies were not used. Such approaches are commonly used to improve DNA vaccine immunogenicity [29], and the relatively inefficient cellular uptake of naked plasmid DNA may therefore have limited the magnitude of vaccine-induced immune responses and contributed to the restricted therapeutic efficacy observed in AAV-HBV mice. The present findings should not be interpreted as representing the maximal immunogenic or therapeutic potential of the DNA-HBsAg-Fc vaccine. This delivery limitation likely acts in conjunction with persistent antigen exposure and the tolerogenic hepatic environment rather than representing the sole explanation for the limited therapeutic efficacy. Future studies should evaluate electroporation and other clinically relevant nucleic-acid delivery approaches to determine whether improved delivery can enhance cellular immunogenicity and therapeutic efficacy. The relatively small group size (n = 5 per group), particularly given the inter-animal heterogeneity observed in some histological outcomes, should be considered when interpreting these findings. Accordingly, the present study should be regarded as an exploratory preclinical study, and larger, prospectively powered studies will be required to confirm the robustness and reproducibility of these observations. In addition, only female C57BL/6J mice were included in the present study. Potential sex- and strain-dependent differences in vaccine immunogenicity and hepatic immune tolerance were therefore not assessed, and the generalizability of these findings to male mice or other genetic backgrounds will require further investigation. Given that mRNA-LNP vaccines bypass the requirement for nuclear entry, enable direct cytoplasmic translation, and provide additional innate immune stimulation through both the mRNA and LNP components [30,31,32], transferring the HBsAg-Fc fusion design to an mRNA-LNP platform warrants further investigation as a strategy to enhance cellular immunogenicity.

Furthermore, the restricted efficacy of vaccine monotherapy underscores the need for combination approaches to achieve a functional cure. We previously proposed a “sandwich” therapeutic strategy for CHB comprising three sequential phases: (1) suppressing HBV replication with antiviral agents; (2) depleting serum HBsAg with potent neutralizing monoclonal antibodies; and (3) upon clearance of serum HBV DNA and HBsAg, deploying active specific immunotherapy (e.g., therapeutic vaccines) to mount durable HBV-specific immune responses against residual HBV [33]. Within this framework, nucleic acid vaccines incorporating the Fc-mediated antigen-targeting strategy could serve as an immune-restorative component of combination regimens. Combining these vaccines with NAs, siRNAs, monoclonal antibodies, or other immunomodulatory agents should be systematically evaluated to determine whether the “sandwich” strategy can more effectively overcome immune tolerance and increase the likelihood of achieving a functional cure.

## 4. Materials and Methods

### 4.1. Animals

Female C57BL/6J mice (6 weeks old) were purchased from Zhejiang Vital River Laboratory Animal Technology Co., Ltd. (Jiaxing, Zhejiang, China) and housed at the Animal Experiment Center of Fudan University. All animal experiments were approved by the Ethics Committee of the Animal Experiment Center of Fudan University (Approval No.: 2022110022S). Mice were maintained under standard environmental and husbandry conditions, and all procedures complied with Chinese national guidelines for laboratory animal care and use.

### 4.2. Cell Culture

HEK293T cells (ATCC, Manassas, VA, USA) were cultured in DMEM (Gibco, Grand Island, NY, USA) supplemented with 10% fetal bovine serum (FBS, Gibco) and 1% penicillin–streptomycin (Meilunbio, Dalian, China). Primary mouse splenocytes were cultured in R10 medium, consisting of RPMI 1640 (Gibco) supplemented with 10% FBS and 1% penicillin–streptomycin. All cells were maintained at 37 °C in a humidified 5% CO_2_ atmosphere.

### 4.3. Vaccine Preparation and In Vitro Characterization

The IC vaccine was produced as reported previously [34]. The coding sequences of DNA-HBsAg and DNA-HBsAg-Fc were codon-optimized, synthesized, and cloned into the pVAX1 plasmid by GenScript Biotech Co., Ltd. (Nanjing, China). Plasmids were transformed into *E*. *coli* DH5α competent cells (Tsingke Biotechnology, Beijing, China), verified by Sanger sequencing, and purified using the EndoFree Plasmid Mega Kit (QIAGEN, Hilden, Germany). For in vitro characterization, plasmids were transfected into HEK293T cells using PolyJet transfection reagent (SignaGen Laboratories, Frederick, MD, USA). Culture supernatants and cell lysates were collected 48 h after transfection for analysis.

### 4.4. Western Blot Analysis

HEK293T cells were harvested 48 h after transfection and lysed in RIPA lysis buffer (Beyotime, Shanghai, China) supplemented with 1 mM PMSF (Beyotime). Protein concentrations of the cell lysates were determined using an Enhanced BCA Protein Assay Kit (Beyotime). Equal amounts of protein (30 μg per lane) were separated on 4–12% Bis-Tris gels (GenScript, Nanjing, China) by SDS-PAGE under reducing conditions and transferred onto PVDF membranes. The membranes were blocked with NcmBlot blocking buffer (NCM, Suzhou, China) for 15 min at room temperature and subsequently incubated overnight at 4 °C with primary antibodies against HBsAg (Novus Biologicals, Centennial, CO, USA, NB100-62652, 1:3000), human IgG1 Fc (ABclonal, Woburn, MA, USA, A21233, 1:2000), or GAPDH (ABclonal, A19056, 1:100,000). After washing, the membranes were incubated with HRP-conjugated goat anti-rabbit IgG (ABclonal, AS013, 1:5000) or HRP-conjugated goat anti-mouse IgG (ABclonal, AS014, 1:5000), as appropriate, for 1 h at room temperature. Protein bands were visualized using an enhanced chemiluminescence reagent (Tanon, Shanghai, China) and imaged with a chemiluminescence imaging system (Tanon).

### 4.5. Vaccination Protocols in Mice

The doses used in this study were selected based on established experimental regimens for the respective vaccine platforms. For immunogenicity studies, C57BL/6J mice were immunized intramuscularly four times at two-week intervals. All vaccine formulations were prepared or diluted with sterile PBS to a final injection volume of 100 μL per mouse. DNA-HBsAg, DNA-HBsAg-Fc, and the empty pVAX1 control were administered at 100 μg plasmid DNA per dose. The rHBsAg and IC vaccines were administered at 2 μg per dose. The rHBsAg vaccine was provided by Kangtai Biological Products Co., Ltd. (Shenzhen, China) and was supplied as an aluminum hydroxide-adjuvanted formulation. Serum and splenocytes were collected two weeks after the final immunization. For the pre-immunization/AAV-HBV transduction model, mice received the same four-dose regimen followed by tail-vein injection of 2 × 10^10^ genome copies (GC) of rAAV8-HBV1.3 (PackGene, Guangzhou, China) two weeks after the final dose. For the AAV-HBV model, mice were first injected intravenously with 2 × 10^10^ GC of rAAV8-HBV1.3 and, seven weeks after transduction, received the same four-dose therapeutic vaccination regimen.

### 4.6. Enzyme-Linked Immunosorbent Assay (ELISA) for Anti-HBs Titers

Serum anti-HBs titers were determined using a commercial ELISA kit (KHB, Shanghai, China). Briefly, two-fold serial serum dilutions starting at 1:25 were incubated in pre-coated microplates for 1 h at 37 °C. Bound antibodies were detected with horseradish peroxidase (HRP)-conjugated goat anti-mouse IgG (Proteintech, Rosemont, IL, USA) for 1 h at 37 °C, followed by chromogenic substrate incubation for 15 min at 37 °C. Absorbance was measured at 450 nm using a microplate reader (Bio-Rad, Hercules, CA, USA). The endpoint titer was defined as the highest serum dilution with an absorbance at least 2.1-fold higher than that of the negative control. The lowest tested serum dilution (1:25) was regarded as the LLoD for anti-HBs endpoint titers. Samples below this limit were assigned a value of 12.5, corresponding to one-half of the reciprocal of the lowest tested dilution, before log_10_ transformation for GMT calculation and graphical presentation.

### 4.7. Quantification of HBsAg and HBV DNA

HBsAg levels in HEK293T-cell lysates, culture supernatants, and mouse serum, as well as serum HBV DNA levels, were measured by KingMed Diagnostics (Shanghai, China). HBsAg was quantified using a commercial HBsAg chemiluminescence immunoassay kit (Maccura Biotechnology Co., Ltd., Chengdu, China), with an LLoD of 0.03 IU/mL. Serum HBV DNA was quantified using a commercial PCR-fluorescent probe HBV nucleic acid detection kit (Daan Gene Co., Ltd., Guangzhou, China), with an LLoD of 100 IU/mL.

### 4.8. IFN-γ Enzyme-Linked Immunospot (ELISpot) Assay

IFN-γ ELISpot assays were performed using a commercial kit (BD Biosciences, San Jose, CA, USA). Briefly, microplates were coated with anti-mouse IFN-γ antibody overnight at 4 °C and blocked with R10 medium for 2 h at room temperature. Splenocytes (1 × 10^6^ cells/well) were stimulated with the HBsAg peptide pool (2 μg/mL), which was provided by Prof. Wenhong Zhang at Fudan University and was identical to that described previously [35]. The peptide pool was designed based on HBV HBsAg of genotype C, serotype adr. Detailed information on the individual peptide sequences, peptide lengths, and corresponding HBsAg regions has been reported in the original publication and its Supplementary Materials. PMA (25 ng/mL) plus ionomycin (1 μg/mL) and medium alone served as positive and negative controls, respectively. After 16 h at 37 °C in 5% CO_2_, spots were developed using a biotinylated detection antibody, streptavidin–HRP, and AEC substrate and counted using an automated ELISpot reader (AID GmbH, Strassberg, Germany).

### 4.9. Intracellular Cytokine Staining (ICS)

Splenocytes (1 × 10^6^ cells/well) were stimulated with the HBsAg peptide pool (2 μg/mL), with medium alone serving as the negative control. After 2 h at 37 °C in 5% CO_2_, GolgiStop (BD Biosciences, San Jose, CA, USA) was added, and incubation continued for an additional 12 h. Cells were stained with a viability dye and surface-marker antibodies, followed by fixation, permeabilization, and intracellular cytokine staining. Samples were acquired on a flow cytometer (Cytek Biosciences, Fremont, CA, USA). Antibody details and gating strategies are provided in Table S1 and Figure S1, respectively.

### 4.10. Immunohistochemistry (IHC)

Liver tissues were fixed in fixative (Servicebio, Wuhan, China), embedded in paraffin, sectioned, and stained with an anti-HBcAg antibody (ZSGB-BIO, Beijing, China). For each animal, one liver section was analyzed. Five fields were randomly selected from each section, and the percentage of HBcAg^+^ hepatocytes in each field was quantified using the histochemistry analysis module of Saiviewer software (version 2.4.0; Servicebio). The mean value of the five fields was used as the representative value for each animal. Field selection and histological quantification were performed blinded to treatment group.

### 4.11. Statistical Analysis

Data were processed and visualized using GraphPad Prism 10.0 (GraphPad Software, San Diego, CA, USA) and are presented as mean ± SEM. Because this was an exploratory preclinical study, no single primary endpoint was prospectively designated, and the immunological and virological readouts were considered exploratory. Group comparisons at experimental endpoints were performed by one-way ANOVA followed by Tukey’s multiple-comparisons test. Longitudinal serum measurements with sufficient quantifiable values were analyzed by two-way repeated-measures ANOVA with Geisser–Greenhouse correction, followed, where appropriate, by Dunnett’s multiple-comparisons test. Longitudinal datasets substantially affected by values below the assay LLoD were summarized descriptively rather than subjected to repeated-measures analysis. Statistical significance was defined as * *p* < 0.05, ** *p* < 0.01, *** *p* < 0.001, and **** *p* < 0.0001; ns, not significant (*p* > 0.05).

## 5. Conclusions

This study evaluated the immune responses and antiviral efficacy of distinct vaccine modalities in naïve mice, a pre-immunization/AAV-HBV transduction model, and a therapeutic AAV-HBV model. The rHBsAg vaccine predominantly induced humoral immunity, and the IC vaccine primarily induced humoral and CD4^+^ T-cell responses, whereas both DNA-HBsAg and DNA-HBsAg-Fc elicited robust CD8^+^ T-cell immunity, which was further augmented by IgG1 Fc fusion. Our results highlight that HBsAg-specific CD8^+^ T-cell responses were associated with greater reductions in intrahepatic HBcAg expression, rather than humoral or CD4^+^ T-cell responses. Nevertheless, in the therapeutic AAV-HBV model, these vaccine monotherapies were insufficient to restore effective HBV-specific T-cell immunity or achieve sustained serum HBsAg clearance. These findings provide insights into the distinct immunological profiles and therapeutic limitations of different vaccine modalities. Future strategies may explore transferring the HBsAg-Fc design to an mRNA-LNP platform or combining such vaccines with antiviral agents to enhance their therapeutic potential.

## Figures and Tables

**Figure 1:**
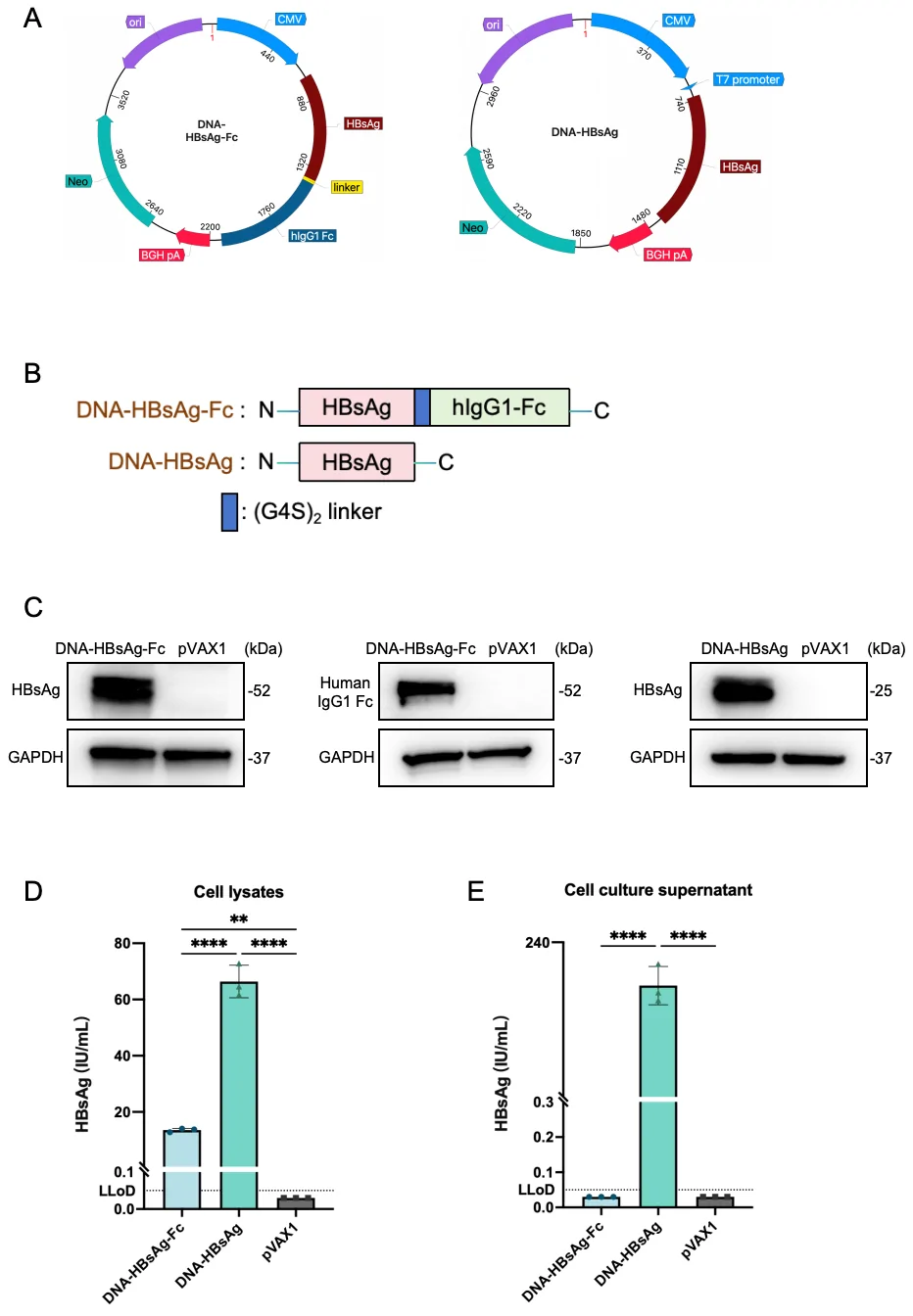
Construction and in vitro characterization of DNA vaccines. (**A**) Schematic maps of the DNA-HBsAg-Fc and DNA-HBsAg vaccine constructs. (**B**) Diagrams of the predicted proteins: DNA-HBsAg encodes the HBsAg; DNA-HBsAg-Fc encodes the HBsAg-human IgG1 Fc fusion protein linked by a (G4S)_2_ linker. (**C**) HEK293T cells were transfected with the indicated plasmids, and cell lysates were analyzed by Western blot at 48 h post-transfection. HBsAg and HBsAg-Fc were detected using an anti-HBsAg antibody, and the Fc component of HBsAg-Fc was additionally detected using an anti-human IgG1 Fc antibody. GAPDH served as the loading control. (**D**,**E**) HBsAg levels in cell lysates (**D**) and culture supernatants (**E**) were quantified using a chemiluminescence immunoassay. Data in (**D**,**E**) are presented as mean ± SEM from three independent experiments. Statistical significance was determined by one-way ANOVA followed by Tukey’s multiple comparisons test. ** *p* < 0.01 and **** *p* < 0.0001.

**Figure 2:**
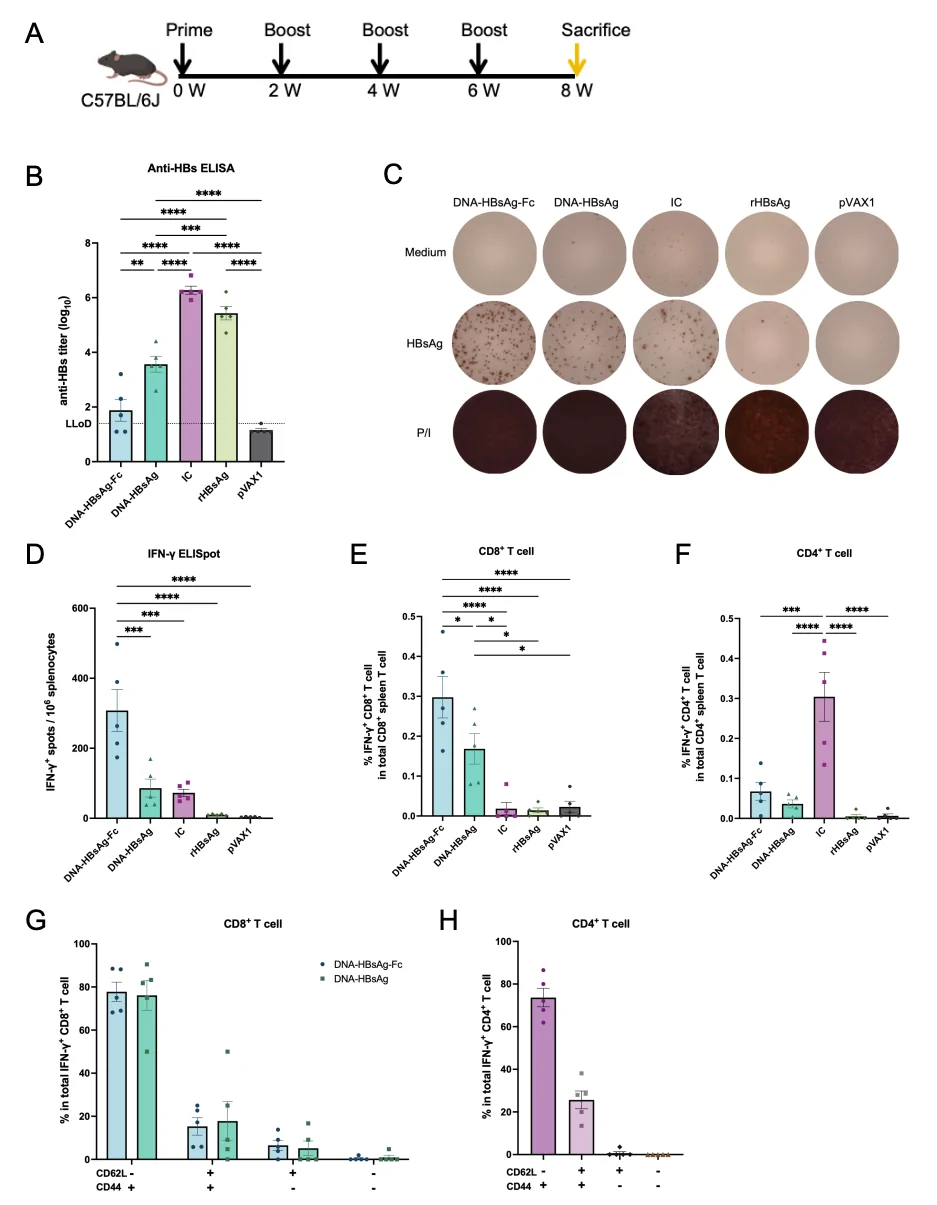
Distinct immune profiles induced by different vaccine modalities in C57BL/6J mice. (**A**) Immunization schedule. Female C57BL/6J mice (n = 5) received intramuscular immunizations at weeks 0, 2, 4, and 6, with immunological analyses performed two weeks after the final dose. (**B**) Serum anti-HBs titers. (**C**) Representative IFN-γ ELISpot images of splenocytes stimulated with the HBsAg peptide pool; PMA/ionomycin and medium alone served as positive and negative controls, respectively. Each image represents an individual mouse from a different group. (**D**) Quantification of IFN-γ SFCs. (**E**,**F**) Frequencies of IFN-γ^+^ CD8^+^ (**E**) and IFN-γ^+^ CD4^+^ (**F**) T cells among splenocytes stimulated with the HBsAg peptide pool, assessed by flow cytometry. (**G**,**H**) Phenotypic subset composition (CD62L^−^ CD44^−^, CD62L^−^ CD44^+^, CD62L^+^ CD44^+^, and CD62L^+^ CD44^−^) of the IFN-γ^+^ CD8^+^ T cells induced by DNA vaccines (**G**) and the IFN-γ^+^ CD4^+^ T cells induced by the IC vaccine (**H**). Phenotypic profiling was restricted to these groups, as the marginal frequency of IFN-γ^+^ cells in the other groups precluded reliable statistical evaluation. Data are presented as mean ± SEM. Statistical significance was determined by one-way ANOVA followed by Tukey’s multiple comparisons test. * *p* < 0.05, ** *p* < 0.01, *** *p* < 0.001, and **** *p* < 0.0001.

**Figure 3:**
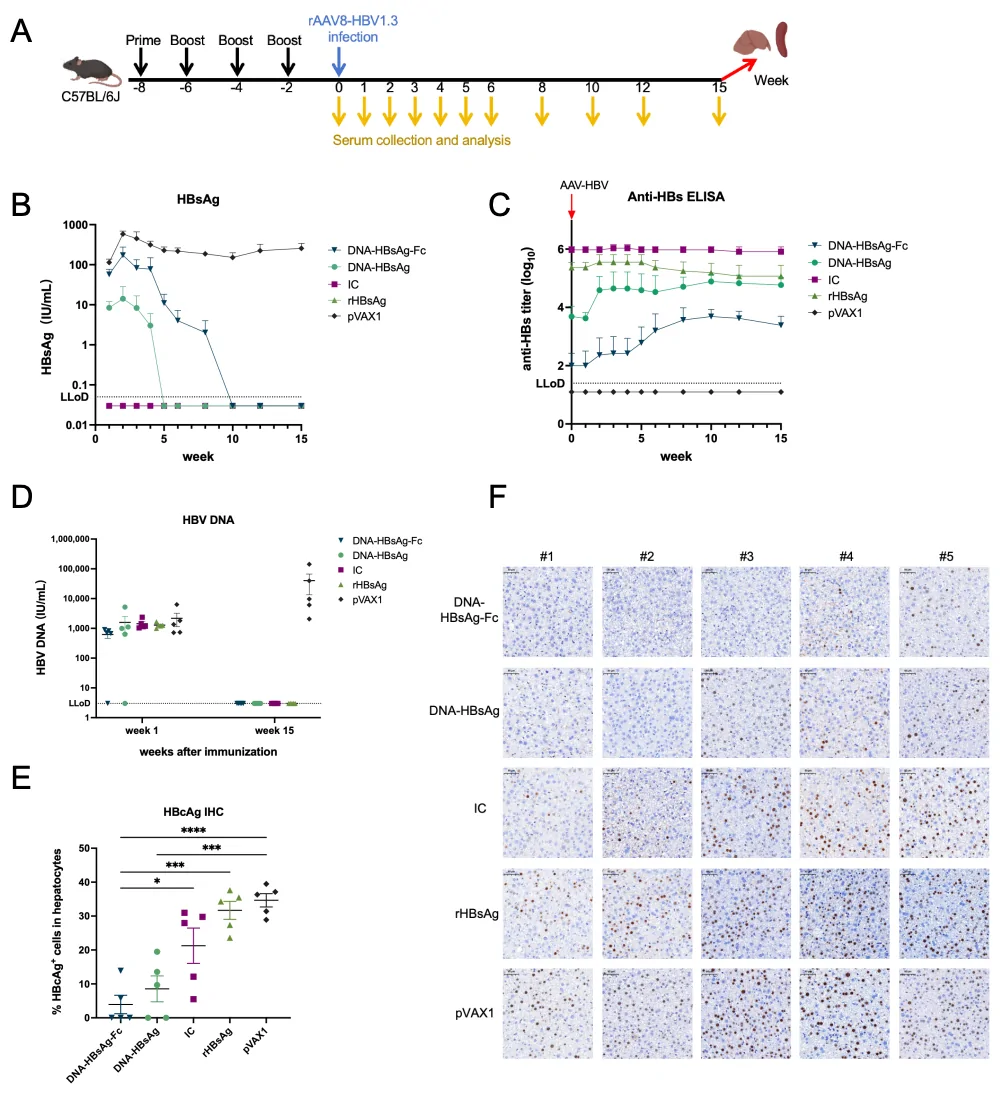
Serological dynamics and intrahepatic HBcAg expression in the pre-immunization/AAV-HBV transduction model. (**A**) Immunization and AAV-HBV transduction schedule. Female C57BL/6J mice (n = 5) received intramuscular immunizations at weeks 0, 2, 4, and 6, and were injected with AAV-HBV via the tail vein two weeks after the final dose. Serum was collected at regular intervals. (**B**,**C**) Kinetics of serum HBsAg (**B**) and anti-HBs titers (**C**) following AAV-HBV transduction. (**D**) Serum HBV DNA levels at week 1 post-transduction and at the observational endpoint. (**E**) Percentage of HBcAg^+^ hepatocytes in liver sections at the observational endpoint. (**F**) Representative HBcAg IHC images of liver sections at the observational endpoint (scale bar = 50 μm). Data are presented as mean ± SEM. Serum HBsAg kinetics in (**B**) were summarized descriptively because several vaccine groups contained values below the assay LLoD throughout or during part of the observation period, precluding reliable repeated-measures analysis of quantitative HBsAg values. For serum anti-HBs kinetics in (**C**), the pVAX1 group remained below the LLoD throughout the observation period and was therefore summarized descriptively, whereas longitudinal anti-HBs responses among the four vaccine groups were analyzed by two-way repeated-measures ANOVA with Geisser–Greenhouse correction. Endpoint comparisons in (**E**) were performed by one-way ANOVA followed by Tukey’s multiple comparisons test. * *p* < 0.05, *** *p* < 0.001, and **** *p* < 0.0001.

**Figure 4:**
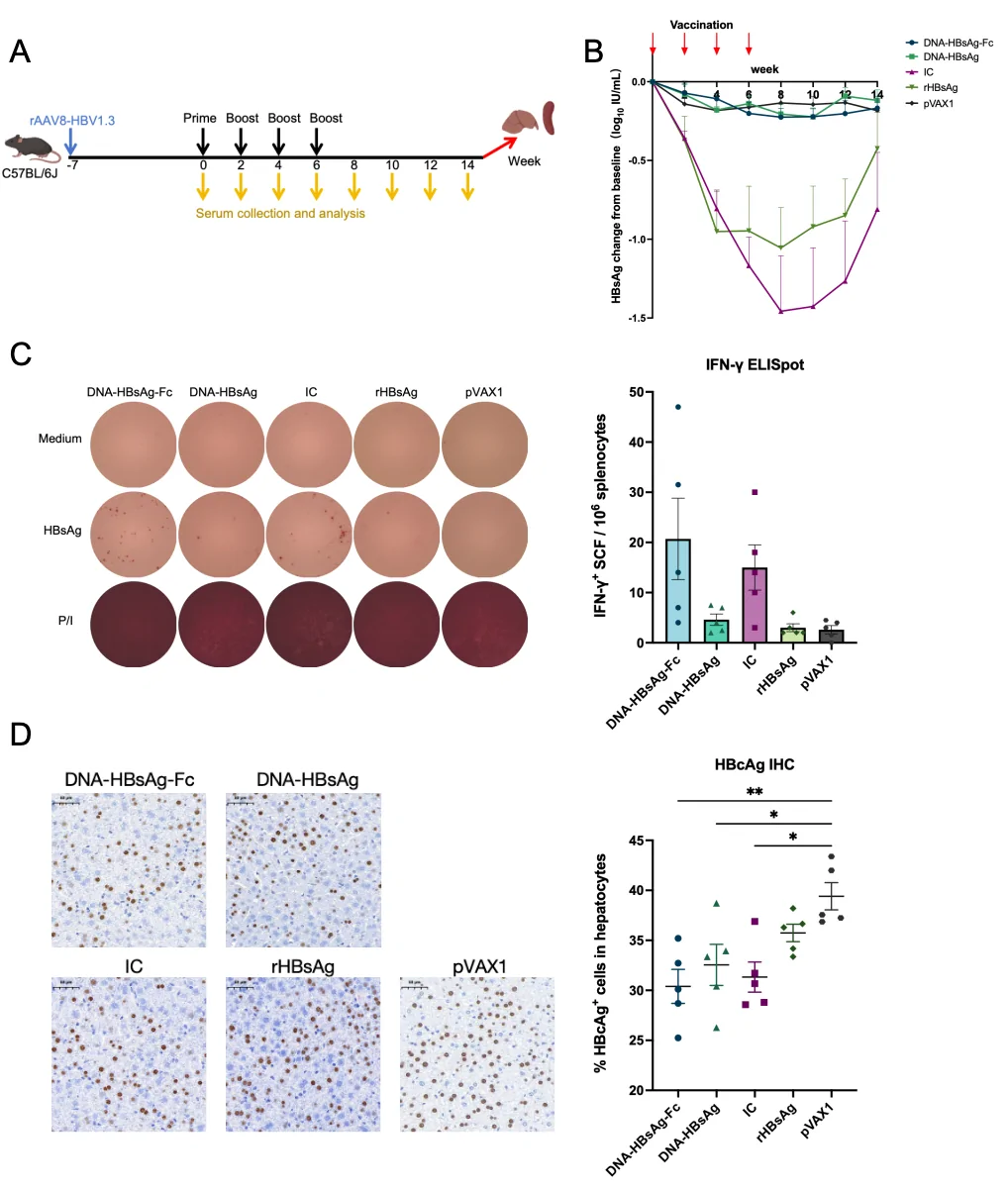
Vaccine monotherapies exhibit limited therapeutic efficacy in AAV-HBV mice. (**A**) Therapeutic schedule. Female C57BL/6J mice (n = 5) were injected with AAV-HBV via the tail vein. Seven weeks after AAV-HBV transduction, therapeutic immunizations were administered four times at two-week intervals, and serum was collected at regular intervals. (**B**) Kinetics of serum HBsAg. (**C**) Representative IFN-γ ELISpot images and quantification of IFN-γ SFCs from splenocytes stimulated with the HBsAg peptide pool. PMA/ionomycin and medium alone served as positive and negative controls, respectively. Each image represents an individual mouse from a different group. (**D**) Representative HBcAg IHC images of liver sections (scale bar = 50 μm) and percentage of HBcAg^+^ hepatocytes at the observational endpoint. Data are presented as mean ± SEM. Longitudinal changes in serum HBsAg from baseline in (**B**) were analyzed by two-way repeated-measures ANOVA with Geisser–Greenhouse correction, followed by Dunnett’s multiple-comparisons test comparing each vaccine group with the pVAX1 control at individual time points. Endpoint comparisons in (**C**,**D**) were performed by one-way ANOVA followed by Tukey’s multiple-comparisons test. * *p* < 0.05, ** *p* < 0.01.

## Data Availability

The original contributions presented in this study are included in the article/Supplementary Materials. Further inquiries can be directed to the corresponding authors.

## Supplementary Materials

The following supporting information can be downloaded at https://www.mdpi.com/article/10.3390/ijms27198499/s1.
