# Supplementary material for: Immune Profiles Elicited by Different HBV Vaccine Modalities and Their Associations with Serological and Intrahepatic Viral Outcomes
Source: Int J Mol Sci. 2026 Sep 23;27(19):8499. doi: 10.3390/ijms27198499 (PMC13664768; doi:10.3390/ijms27198499)
Supplement: Supplementary file 1 [file ijms-27-08499-s001.zip › ijms-4565272-supplementary.pdf]

## Supplemental Information

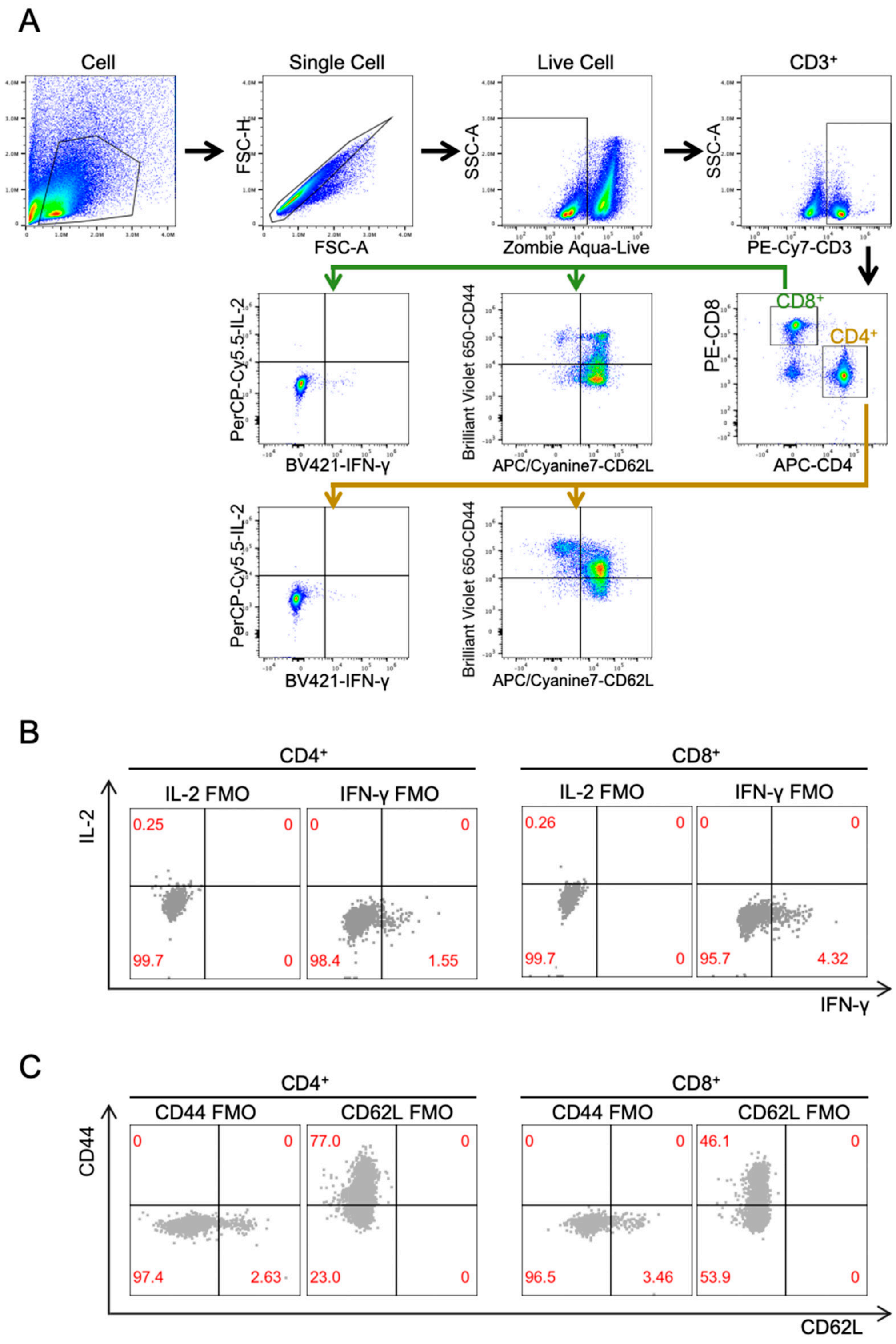


**Figure S1. Gating strategy and fluorescence-minus-one (FMO) controls for flow cytometry.** (A) Gating strategy for ICS analysis. (B, C) Representative FMO controls for IFN-γ and IL-2 (B) and CD44 and CD62L (C) in mouse splenocytes stimulated with PMA and ionomycin.

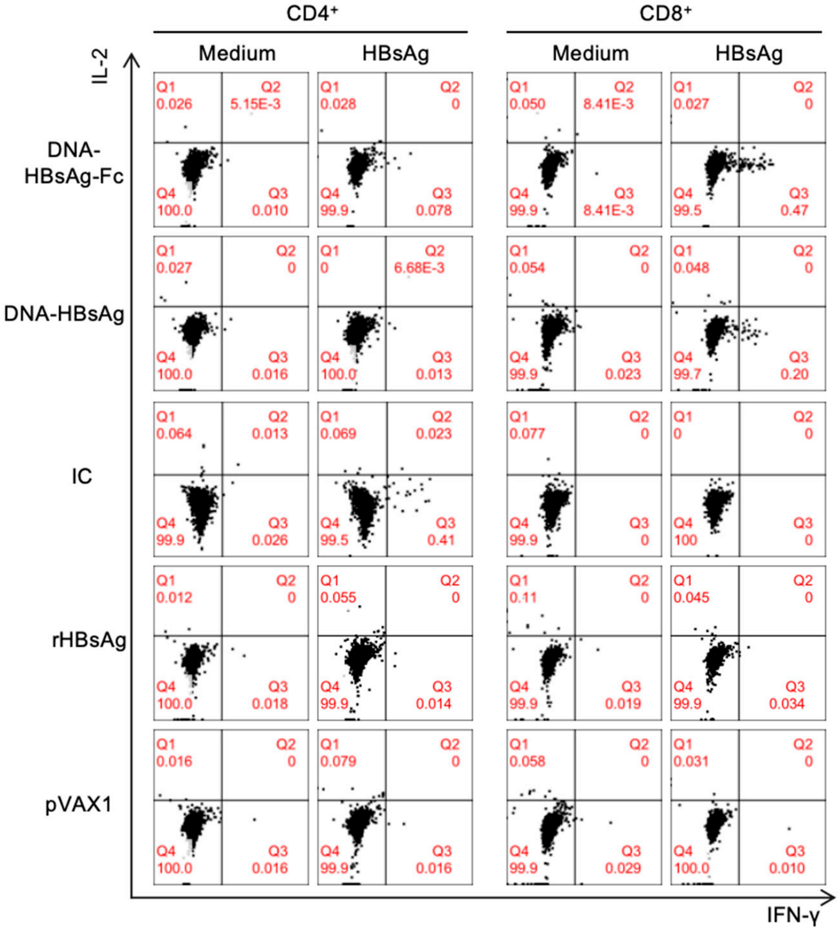


**Figure S2. Flow cytometric analysis of intracellular IFN-γ and IL-2 expression in CD4+ and CD8+ T cells.** Representative flow cytometry dot plots showing intracellular IFN-γ and IL-2 expression in CD4+ and CD8+ T cells in splenocytes from mice in the different vaccine groups following stimulation with the HBsAg peptide pool. Medium alone served as the negative control.

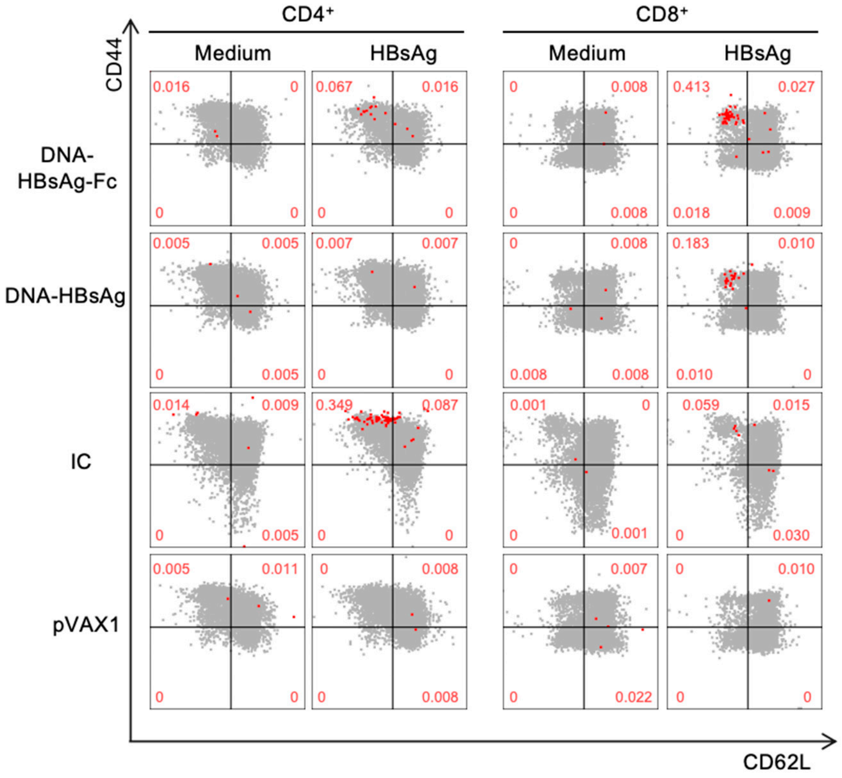


**Figure S3. Expression profiles of CD44 and CD62L on IFN-γ$^+$ T cells.** Representative overlaid flow cytometry dot plots showing the expression of CD44 and CD62L on total CD4$^+$ or CD8$^+$ T cells (gray) versus IFN-γ$^+$ CD4$^+$ or IFN-γ$^+$ CD8$^+$ T cells (red) in splenocytes from responsive groups following stimulation with the HBsAg peptide pool.

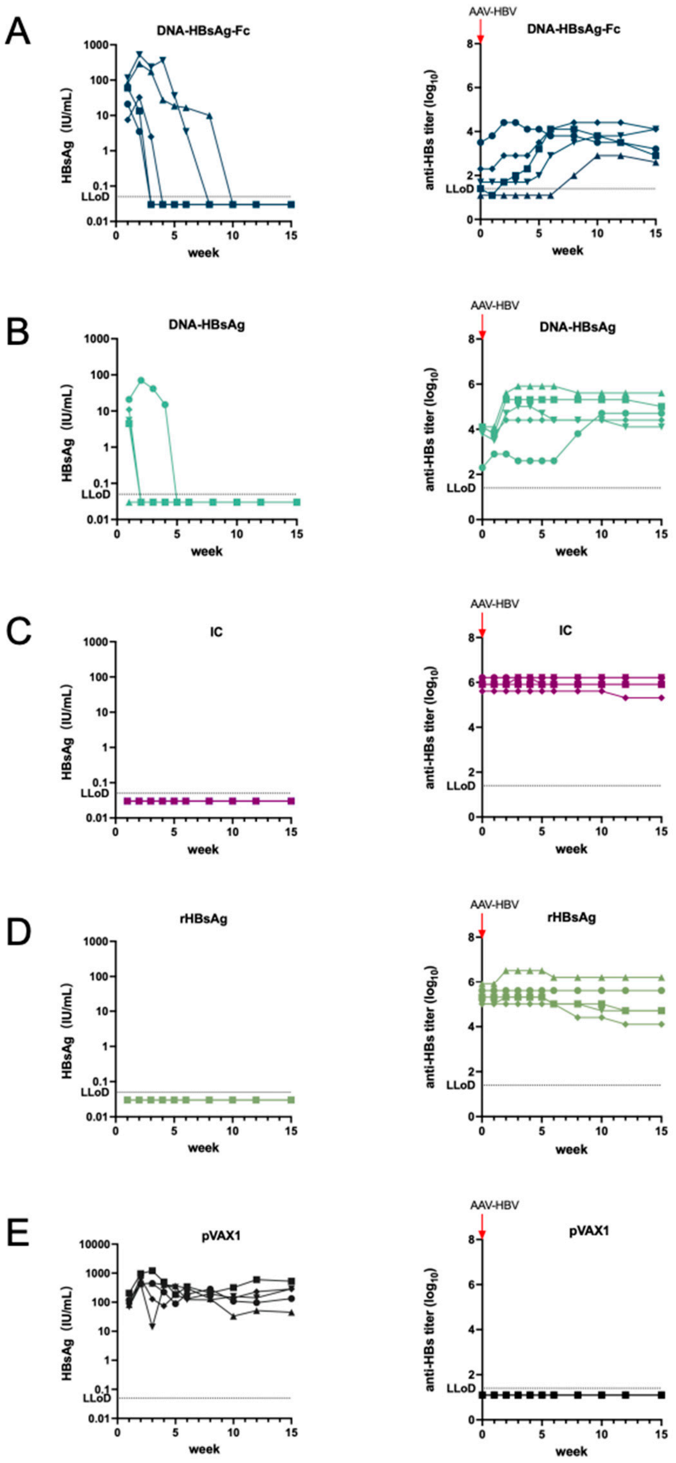


**Figure S4. Serum HBsAg and anti-HBs kinetics in the pre-immunization/AAV-HBV transduction model.** (A-E) Kinetics of serum HBsAg (left) and anti-HBs titers (right) in individual mice immunized with DNA-HBsAg-Fc (A), DNA-HBsAg (B), IC (C), rHBsAg vaccine (D), or empty pVAX1 (E) following AAV-HBV transduction. Each line represents an individual mouse.

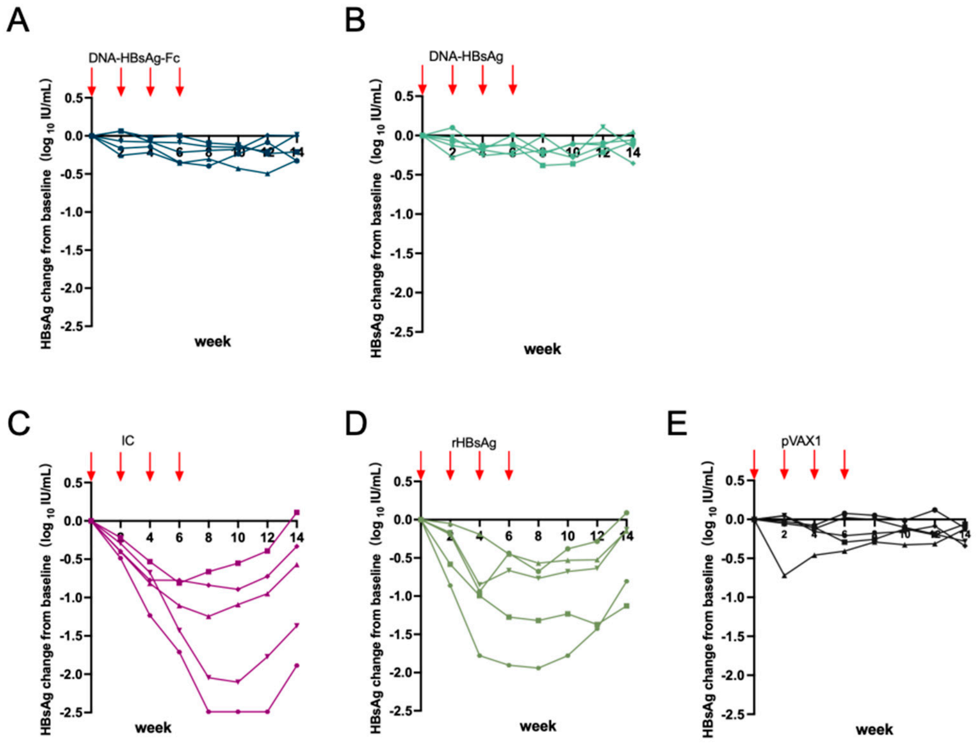


**Figure S5. Serum HBsAg kinetics in AAV-HBV mice.** (A-E) Kinetics of serum HBsAg in individual AAV-HBV mice therapeutically treated with DNA-HBsAg-Fc (A), DNA-HBsAg (B), IC (C), rHBsAg vaccine (D), or empty pVAX1 (E). Each line represents an individual mouse.

**Table S1. Flow cytometry antibody.**

| Name | Supplier | Cat. No. |
|---|---|---|
| Zombie Aqua Fixable Viability Kit | BioLegend | 423101 |
| PE/Cyanine7 anti-mouse CD3 antibody | BioLegend | 100220 |
| APC anti-mouse CD4 antibody | BioLegend | 100412 |
| PE anti-mouse CD8a antibody | BioLegend | 100708 |
| APC/Cyanine7 anti-mouse CD62L antibody | BioLegend | 104428 |
| Brilliant Violet 650 anti-mouse CD44 antibody | BioLegend | 103049 |
| PerCP/Cyanine5.5 anti-mouse IL-2 antibody | BioLegend | 503822 |
| Brilliant Violet 421 anti-mouse IFN-γ antibody | BioLegend | 505830 |

**Table S2. Dunnett-adjusted *p* values for serum HBsAg comparisons between vaccine groups and the pVAX1 control over time in the therapeutic AAV-HBV model.**

| P value / Week / Item | 2 | 4 | 6 | 8 | 10 | 12 | 14 |
|---|---|---|---|---|---|---|---|
| DNA-HBsAg-Fc | 0.9748 | 0.7499 | 0.9897 | 0.7040 | 0.6961 | 0.9203 | 0.9992 |
| DNA-HBsAg | 0.9834 | > 0.9999 | 0.9977 | 0.8520 | 0.6578 | 0.9693 | 0.8613 |
| IC | 0.4923 | **0.0099** | **0.0079** | 0.0504 | 0.0674 | 0.1057 | 0.3727 |
| rHBsAg | 0.6627 | 0.0967 | 0.1280 | 0.0572 | 0.1015 | 0.0919 | 0.7169 |

*p* values were obtained from Dunnett's multiple-comparisons test following two-way repeated-measures ANOVA with Geisser–Greenhouse correction. Each vaccine group was compared with the pVAX1 control at the corresponding time point. Adjusted $p < 0.05$ was considered statistically significant.
